# Dr. Alfred Cleveland Cotton

**Published:** 1916-09

**Authors:** 


**Dr. Alfred Cleveland Cotton,** Rush 1878, a well known paediatrist of Chicago, died at his home July 12, aged 69. He was the author of several books on diseases and dietetic management of children.

